# To investigate the mechanism of Yiwei Decoction in the treatment of premature ovarian insufficiency-related osteoporosis using transcriptomics, network pharmacology and molecular docking techniques

**DOI:** 10.1038/s41598-023-45699-8

**Published:** 2023-11-03

**Authors:** Weisen Fan, Yan Meng, Jing Zhang, Muzhen Li, Yingjie Zhang, Xintian Qu, Xin Xiu

**Affiliations:** 1 First Clinical College of Medicine, Shandong University of Traditional Chinese Medicine, Jinan, 250013 China; 2 School of Health, Shandong University of Traditional Chinese Medicine, Jinan, 250013 China; 3 College of Acupuncture and Massage, Shandong University of Traditional Chinese Medicine, Jinan, 250013 China

**Keywords:** Neuroendocrine diseases, High-throughput screening, Molecular medicine

## Abstract

To investigate the molecular mechanism of Yiwei Decoction (YWD) in preventing Premature ovarian insufficiency (POI)-related osteoporosis from the hypothalamic perspective , and to screen for the key active and acting molecules in YWD. Cyclophosphamide was used to create the POI rat model. Groups A, B, and C were established. The Model + YWD group was group A, the model control group was group B, and the normal control group was group C. ELISA was used to determine serum GnRH and FSH levels after gavage. The transcription levels of mRNAs in each group's hypothalamus tissues were examined using RNA-seq sequencing technology. The GSEA method was used to enrich pathways based on the gene expression levels of each group. The TCM–active ingredient–target–disease network map was created using differentially expressed mRNAs (DEmRNAs) and network pharmacology. The molecular docking method was employed to investigate the affinity of the active ingredient with key targets. GnRH and FSH levels in POI rats' serum were reduced by YWD. Between groups A and B, there were 638 DEmRNAs (P < 0.05) and 55 high-significance DEmRNAs (P-adjust < 0.01). The MAPK, Hedgehog, Calcium, and B cell receptor pathways are primarily enriched in DEmRNAs from Group A and Group B. The GSEA pathway enrichment analysis indicates that YWD may regulate Long-term potentiation, Amphetamine addiction, and the Renin-angiotensin system and play a role in preventing osteoporosis. The Chinese herbal medicine (CHM)—Active ingredient-Target-disease network map includes 137 targets, 4 CHMs, and 22 active ingredients. The result of docking indicated that Stigmasterol, interacts well with the core proteins ALB, VCL and KAT5. Following the screening, we identified the targets, active components, and key pathways associated with YWD osteoporosis prevention. Most of these key targets and pathways are associated with osteoporosis, but further experimental validation is required.

## Introduction

Premature ovarian insufficiency (POI) is a female reproductive endocrine disease, the main clinical manifestations of infertility, abnormal menstruation, hot flashes, decreased sexual desire, etc^1^. Because POI can lead to decreased estrogen in the body, patients with POI often have a loss of bone mass, leading to osteoporosis^2^. The hypothalamus can control the pituitary gland, which controls the ovaries, thyroid, and adrenal glands. As a result, the hypothalamus significantly affects the regulation of female endocrine and metabolic processes. It can function as a regulatory center to control this disease via the neuroendocrine regulatory axis^2^. The hypothalamus is currently a hot topic in osteoporosis research. It has been demonstrated that the hypothalamic arcuate nucleus can regulate bone metabolism^3^, particularly its AgRP, which expresses Agouti-related protein and thus directly participates in bone regulation^4,5^.

In the treatment of osteoporosis, traditional Chinese medicine has the advantages of a good curative effect and few side effects^6^. Chinese herbal medicine has great potential in the treatment of osteoporosis, such as reducing bone loss^7^, promoting osteoblast formation while inhibiting osteoclast formation^8^, balancing bone metabolism, and improving bone density^9^. Yiwei Decoction (YWD) is an effective treatment for Yangming weakness, closely related to osteoporosis^10^. In treating POI, YWD has a positive clinical effect^11^. Transcriptomics was thus used to detect gene expression changes in the hypothalamus following YWD intervention in POI rats to identify key targets for the prevention of POI-related osteoporosis and active ingredients that can play a therapeutic role.

## Materials and methods

### Experimental animals and reagents

The 45 female Sprague Dawley (SD) rats used in this experiment were eight weeks old and provided by Beijing Vitonglihua Experimental Animal Technology Co., LTD. The rats were fed adaptively after entering the barrier environment for one week. Beijing Keao supplied the rat maintenance feed (10 kg/bag). After using the crystal violet staining method to stain the rats' vaginal smears and observe them for ten days in a row, 45 rats were randomly divided into groups A, B, and C after confirming that each rat had a normal estrous cycle.

The Animal Ethics Committee of Shandong University of Traditional Chinese Medicine approved this experiment (SDUTCM20210224024). We strictly follow the animal welfare ethical guidelines (2018 edition) to ensure that the experiments comply with the morality norms and ethical requirements. All experimental steps during the experiment were carried out in accordance with the relevant provisions of the methodology section and ARRIVE guidelines^12^.

Cyclophosphamide (CTX) was used to form groups A and B. The modeling procedure was as follows: CTX50mg/kg was injected for the first time, and a maintenance dose of 8 mg/kg/d was injected continuously for 15 days for modeling^13^. As a control, Group C received an equal volume of normal saline. After modeling, the estrous cycle of the A and B group was observed for ten days using crystal violet staining (source leaf: BYBZ14829). The modeling was successful when the estrous cycle of rats was abnormal for ten consecutive days.

### Experimental animals and specimens collection

Composition of the drug: Glehnia littoralis 18 g, Ophiopogon Japonicus 30 g, Rehmannia glutinosa 30 g, Polygonatum Odoratum 9 g.The Pharmacy Department of Shandong University of Chinese Medicine's Affiliated Hospital provided intragastric Chinese herbs. The drug concentration was 0.87 g/ml, and the herbal compound was concentrated at 100. According to the equivalent dose ratio table of human and animal body surface area, the dose of YWD for rats should be 7.830 g/kg/d. For YWD, Groups A and B received gavage, whereas Group C received gavage with an equal volume of normal saline for four weeks. All rats were sedated for 5 min by inhaling 2% isoflurane (Ryward: HYT11281368). The rats were immobilized after anesthesia, and blood from the hypothalamus and abdominal aorta were removed.

### Transcriptomic analysis

#### RNA extraction

Total RNA was extracted from tissue using Plant RNA Purification Reagent (Invitrogen) according to the manufacturer's instructions, and genomic DNA was removed using DNase I RNase-free (TaKara). The ND-2000 and a 2100 Bioanalyzer (Agilent Technologies, Santa Clara, CA, USA) were used to assess RNA quality (NanoDrop Technologies). Only high-quality RNA samples (OD260/280 = 1.82–2, OD260/230 = 2.0–2.2, RIN8, 28S:18S1.0, > 10 g) were used to build the sequencing library.

#### Library preparation

RNA-seq transcriptome strand library was prepared using 5 g of total RNA using Illumina's TruSeqTM stranded total RNA Kit (San Diego, CA). To summarize, the Ribo-Zero Magnetic kit was used to deplete ribosomal RNA (rRNA) instead of poly (A) purification and then fragmented by fragmentation buffer first. Then, using random hexamer primers, first-stranded cDNA was synthesized. The RNA template was removed, and a replacement strand was synthesized with dUTP instead of dTTP to generate ds cDNA. Because the polymerase did not incorporate past this nucleotide, incorporating dUTP quenched the second strand during amplification. The ds cDNA was separated from the second strand reaction mix using AMPure XP beads. To prevent the blunt fragments from ligating during adapter ligation, an 'A' nucleotide was added to the 3' ends.. Finally, the ends of the ds cDNA were ligated with multiple indexing adapters. On 2% Low Range Ultra Agarose, libraries were size selected for cDNA target fragments of 200–300 bp, which were then PCR amplified for 15 PCR cycles with Phusion DNA polymerase (NEB). Following TBS380 quantification, the paired-end RNA-seq sequencing library was sequenced with the Illumina HiSeq xten/NovaSeq6000 (2 150 bp read length). Furthermore, TruseqTM Small RNA sample prep Kit was used to ligate 3 g of total RNA with sequencing adapters (Illumina, San Diego, CA, USA). Then, cDNA was synthesized via reverse transcription and amplified via 12 PCR cycles to create libraries. Shanghai Majorbio Bio-Pharm Biotechnology Co., Ltd. performed deep sequencing after TBS380 quantification (Shanghai, China).

#### Transcriptome assembly and read mapping

The raw paired-end reads were trimmed and quality controlled using the default parameters of SeqPrep (https://github.com/jstjohn/SeqPrep) and Sickle (https://github.com/najoshi/sickle). The clean reads were aligned to the reference genome in orientation mode using the HIASAT (https://ccb.jhu.edu/software/hisat2/index.shtml) software^14^. StringTie (https://ccb.jhu.edu/software/stringtie/index.shtml?t=example) was used to assemble the mapped reads of each sample in a reference-based approach^15^.

#### Differential expression analysis

To distinguish differentially expressed mRNAs (DEmRNAs) between two samples, the expression level of each transcript was calculated using the TPM method. To calculate gene abundances, RSEM (http://deweylab.biostat.wisc.edu/rsem/) was used^16^. DEmRNAs were extracted using DEseq2 with |log2FC|> 1 and p-value < 0.05^17^.

### ELISA testing

Six abdominal aortic blood samples were drawn randomly from each of the three groups and placed in a centrifuge tube for natural coagulation at room temperature for 15 min. The supernatant was collected after centrifugation at 3000 r/min for 20 min. Complete the GnRH and FSH detection as directed by the merchant.

### Network pharmacology and data analysis

DEmRNAs (P < 0.05) between groups A and B are depicted using volcanic maps. Enter these DEmRNAs into David's website and create a KEGG bubble diagram. To calculate the protein interaction relationship, enter these DEmRNAs into the String website (https://cn.string-db.org/). In Cytoscape3.8.2, open the TSV file and plot the core protein network based on the degree. P-adjust can help to avoid false positives. Heat maps were utilized to display all high-significance DEmRNAs (with P-adjust < 0.01) in order to screen important targets. The GSEA pathway enrichment method was used to analyze the gene expression levels of each group, and the pathway was arranged from small to large based on the P value.

Active Chinese herbal medicine (CHM) ingredients were chosen from the BATMAN—TCM database (http://bionet.ncpsb.org.cn/batman-tcm/index.php). The corresponding target was found based on the active ingredient, and the duplication was removed. Download postmenopausal osteoporosis (PMOP)-related targets from the GeneCards website (https://www.genecards.org/). Venny2.1.0 was used to plot the intersection of PMOP-associated targets, YWD targets, and DEmRNAs between groups A and B. The intersection target was chosen to draw the network diagram of CHM—active ingredient-target-disease. The active ingredient with the most targets was chosen for docking verification with the core protein. The active ingredient is obtained from the PubChem compound database (https://pubchem.ncbi.nlm.nih.gov/), and the protein structure is obtained from the PDB (http://www.rcsb.org/). The proteins that were chosen all belonged to the species Homo sapiens. First, we obtain the active ingredient from the PubChem compound database (https://pubchem.ncbi.nlm.nih.gov/) and the protein structure from the RCSB Protein Data Bank (http://www.rcsb.org/). The removal of water molecules and protein ligands was then carried out using PyMol software, followed by the energy minimization of the small molecules using Chem3D software. Molecular docking research was conducted using Autodock Vina 1.2.2 (http://autodock.scripps.edu/). The docking results were shown in 2D and 3D models using Discovery Studio 2016.

### Molecular dynamics simulation

The molecular dynamics (MD) simulation was carried out using GROMACS 2020.3 software^18,19^. The related topology is first generated by processing the target protein's (the receptor's) file. SwissParam was then utilized to treat Stigmasterol. A complex information file is created by combining the information on small molecules with the protein topology file. A cube with a size of 1.2 was used as the limiting box to calculate the binding scenario, and water was added as the solvent. The basis for this calculation was the TIP3P water model and the charmm 36 force field. Addition of Na + /Cl ion pairs to the system neutralized the charge. To minimize arbitrary contacts and atomic overlap, the system underwent 5.0 × 10^4^ energy optimizations using the steepest descent approach before the MD simulation. Following energy minimization, the NVT ensemble was used to equilibrate the system temperature at 300 K and 100 ps. Pressure equilibration was accomplished using a simulation of a 100 ps NPT set and 1 bar. Presimulation's major objective is to maximize the interaction of the target protein with the solvent and ions to fully preequilibrate the simulated system. After that, the MD simulations were run for 50 ns at 300 K and 1 atmosphere of pressure. Following the MD, the Molecular Mechanics/Generalized Born Surface Area (MMGBSA), radial distribution function (RDF), and root mean square variance (RMSD) of each amino acid trajectory were computed.

## Results

### YWD prevention of POI-related osteoporosis targets and pathways

**Figure 1:**
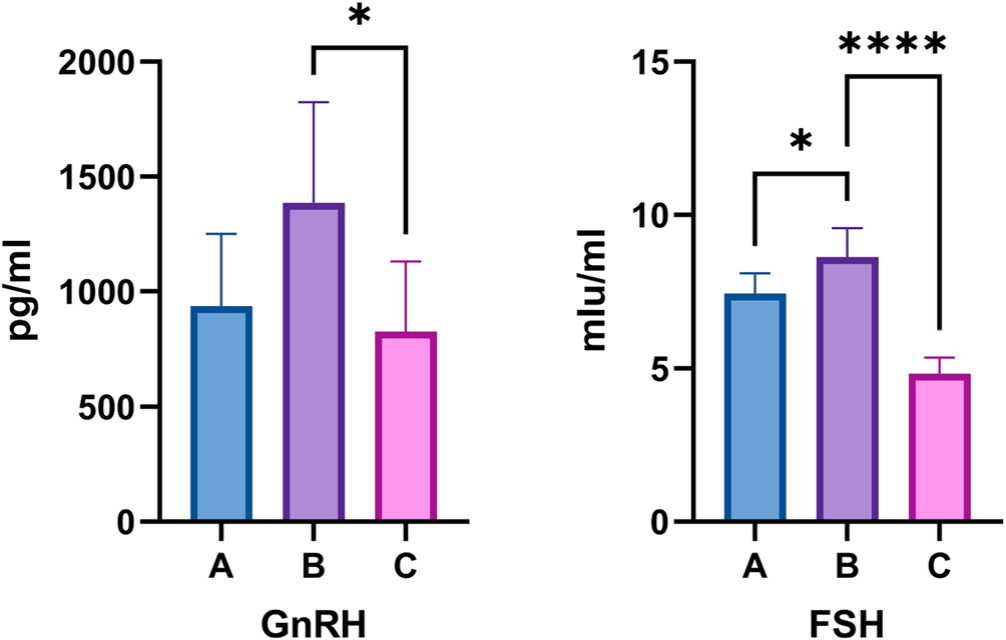
Serum levels of GnRH and FSH in the three groups of SD rats. Serum GnRH and FSH levels were highest in group B and lowest in group C.

**Figure 2:**
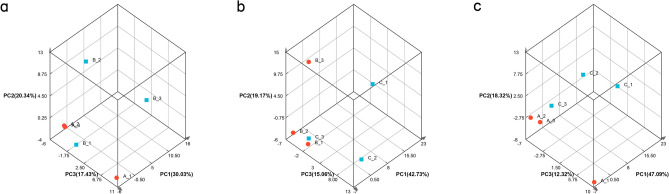
Spatial distribution of transcriptomics principal component analysis. Pairwise comparison analysis of principal components of transcriptomics in groups A, B and C.

**Figure 3:**
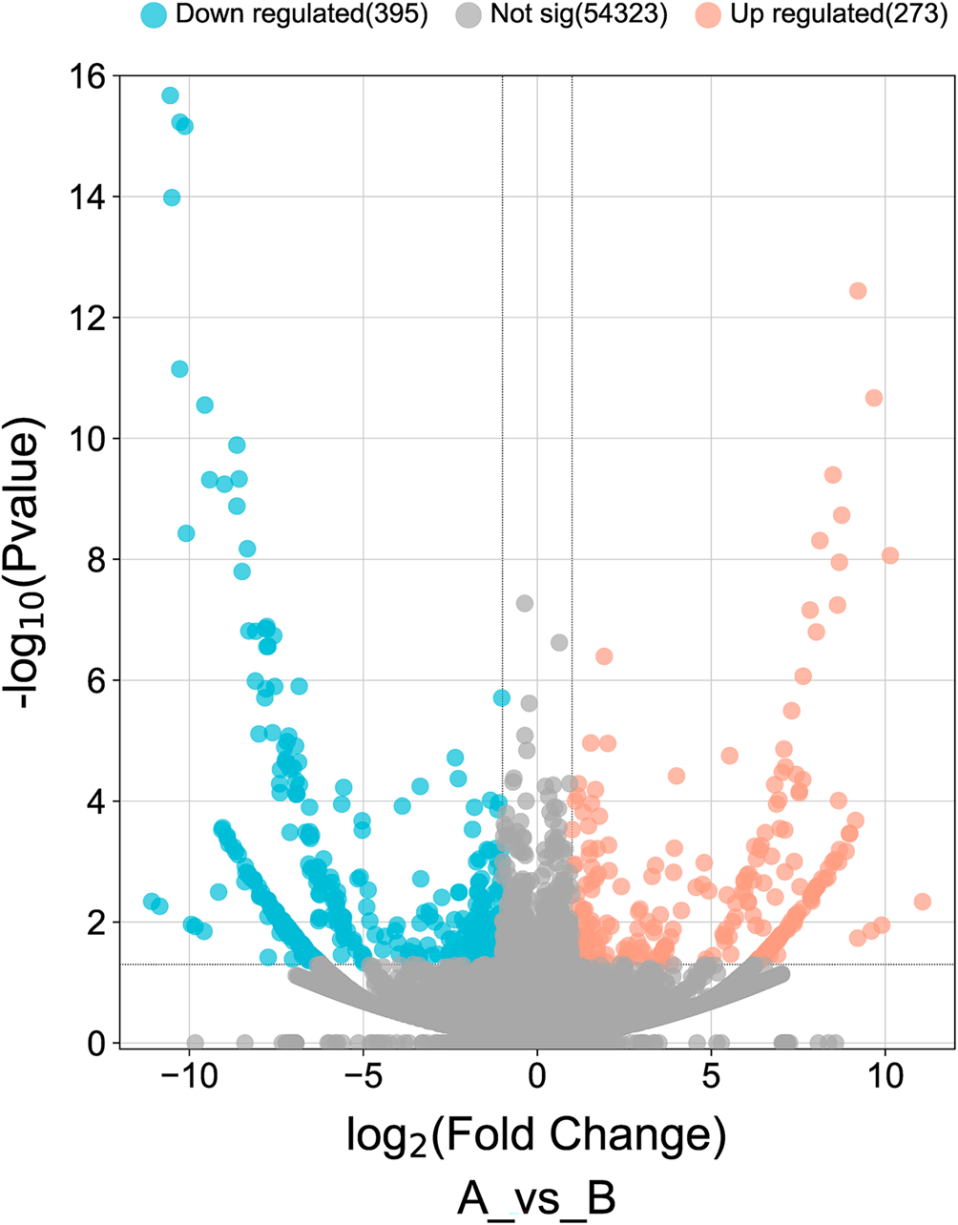
Map of the volcanic distribution of DemRNAs. MRNAs differentially expressed in POI rats following YWD intervention, with red representing up-regulation and blue representing down-regulation.

**Figure 4:**
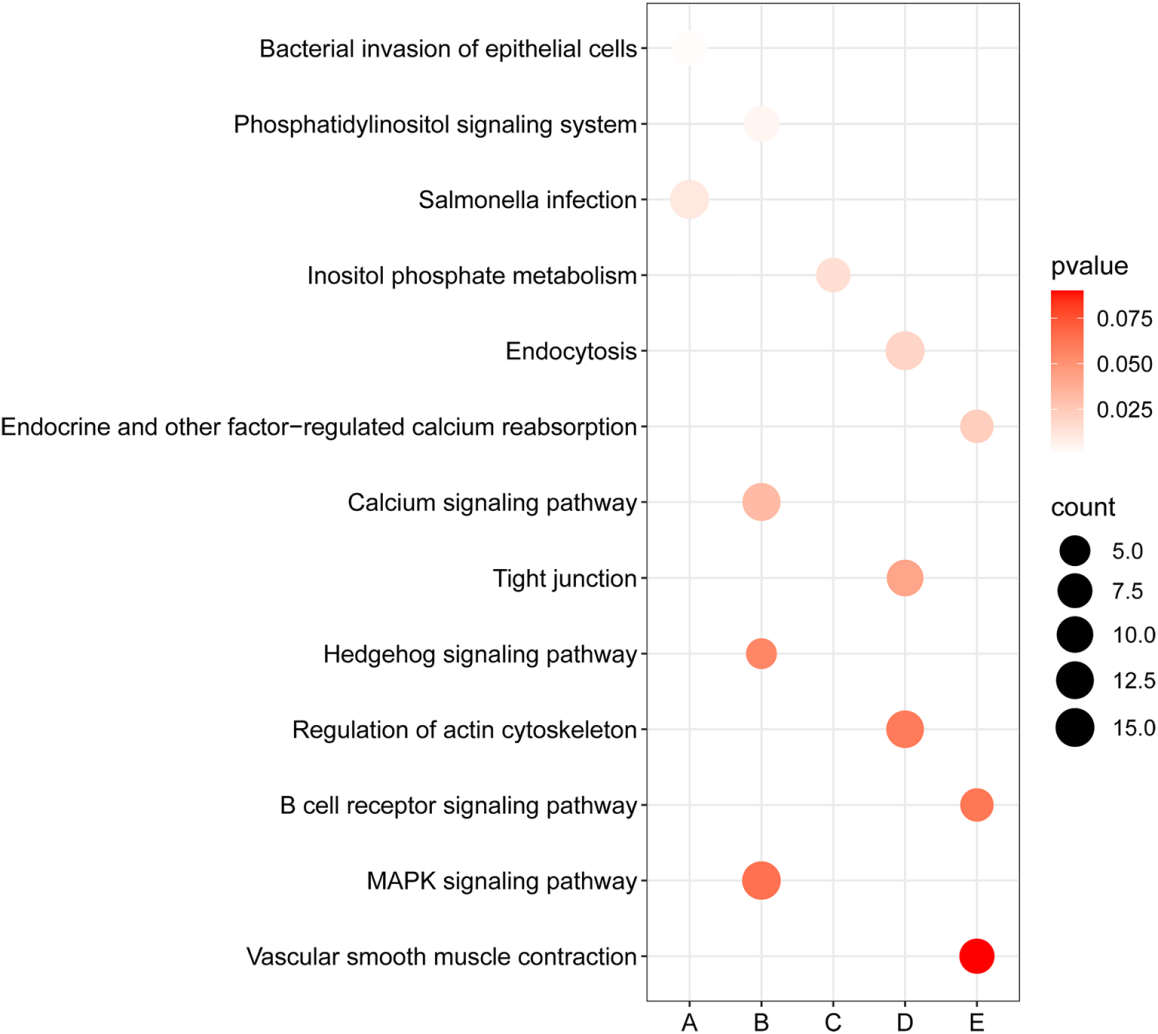
DEmRNA-enriched pathways between groups A and B. The bubble color represents the P value; the lighter the color, the greater the confidence and the bubble size represents the number of DEmRNAs enriched in the pathway. The path type is represented by the letters on the X-axis. (**A**) Diseases of man; (**B**) environmental data processing; (**C**) metabolism; (**D**) cellular procedures; (**E**) organic systems.

Figure 1 shows that GnRH and FSH levels in POI rats' serum are higher than in normal rats, whereas YWD can reduce both levels in POI rats' serum. PCA analysis of the two groups in Fig. 2 showed that the groups were well distinguished. Following the sequencing analysis, we discovered 668 DEmRNAs between groups A and B. Enrichment analysis of KEGG pathways yielded 13 pathways. Figures 3 and 4 show more information. Supplement S1 shows the expression of mRNAs in groups A, B, and C.

### Key target screening

**Figure 5:**
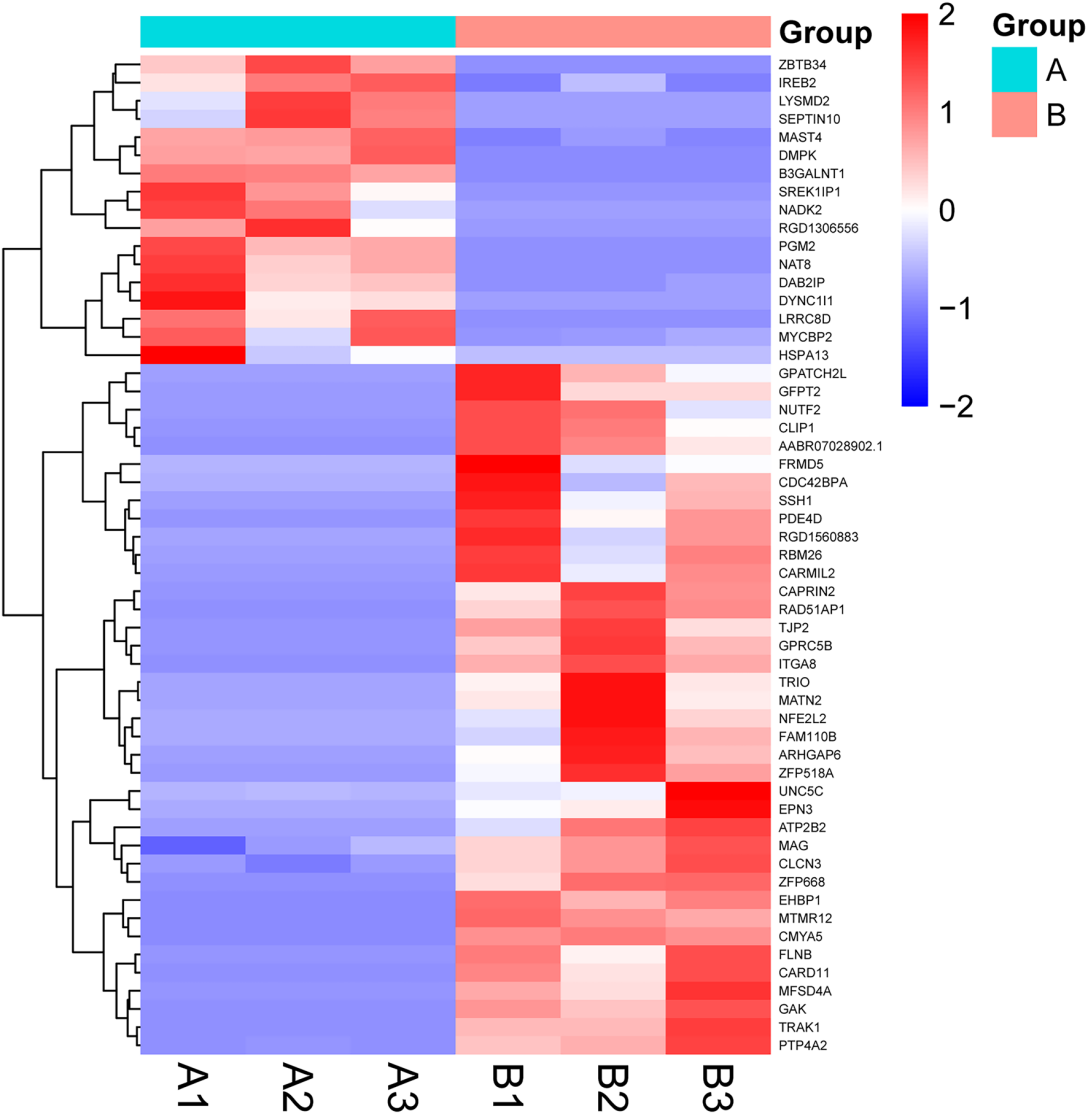
Key target heat map, with red representing high expression and blue representing low expression.

**Figure 6:**
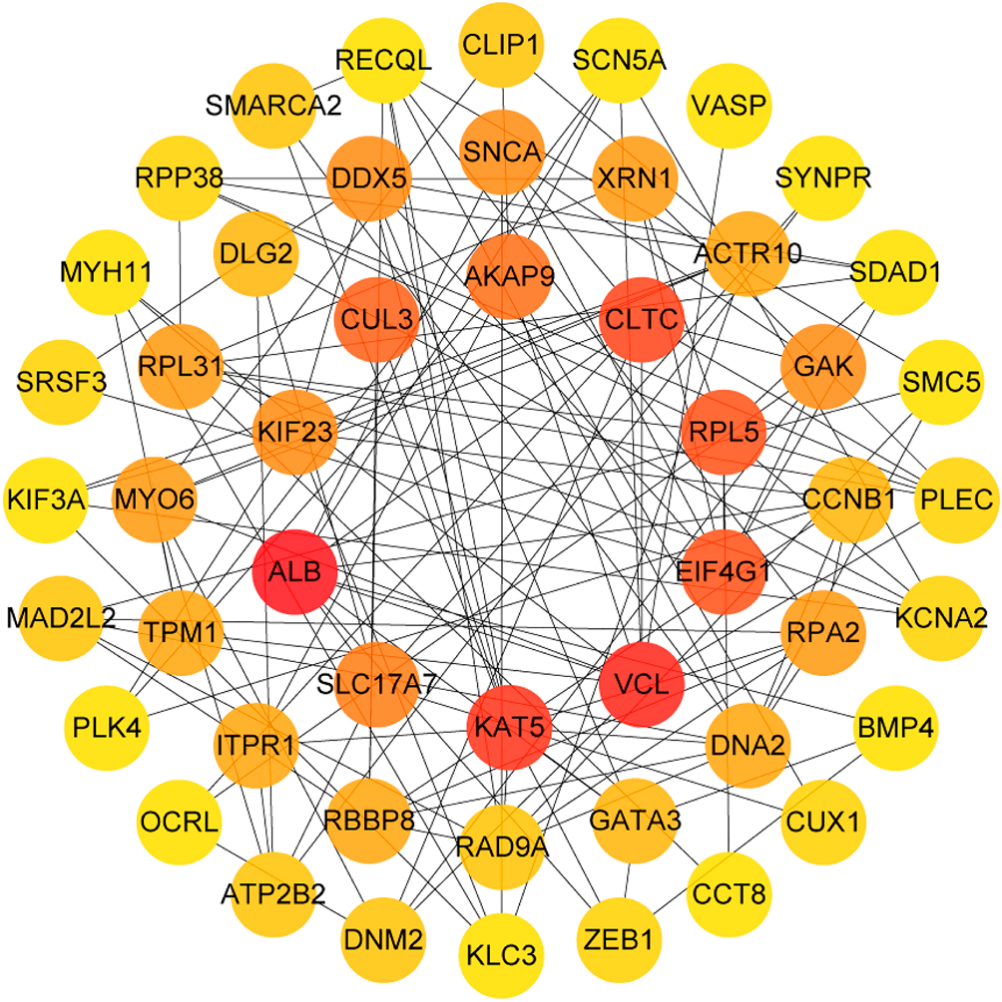
Protein interaction diagram: the darker the color, the higher the Degree value. The lines between targets represent protein interactions.

Between groups A and B, 55 DEmRNAs were obtained after the filter was set to P-adjust < 0.01. For more information, see Fig. 5. The David database was used to calculate the protein interactions between DEmRNA groups A and B, and Cytoscape was used to display the top 30 core proteins. Figure 6 depicts the core proteins of protein interaction, which include ALB, VCL, CLTC, KAT5, and EIF4G1, among others. Supplement S2 depicts the protein interaction.

### Analysis of GSEA enrichment

**Figure 7:**
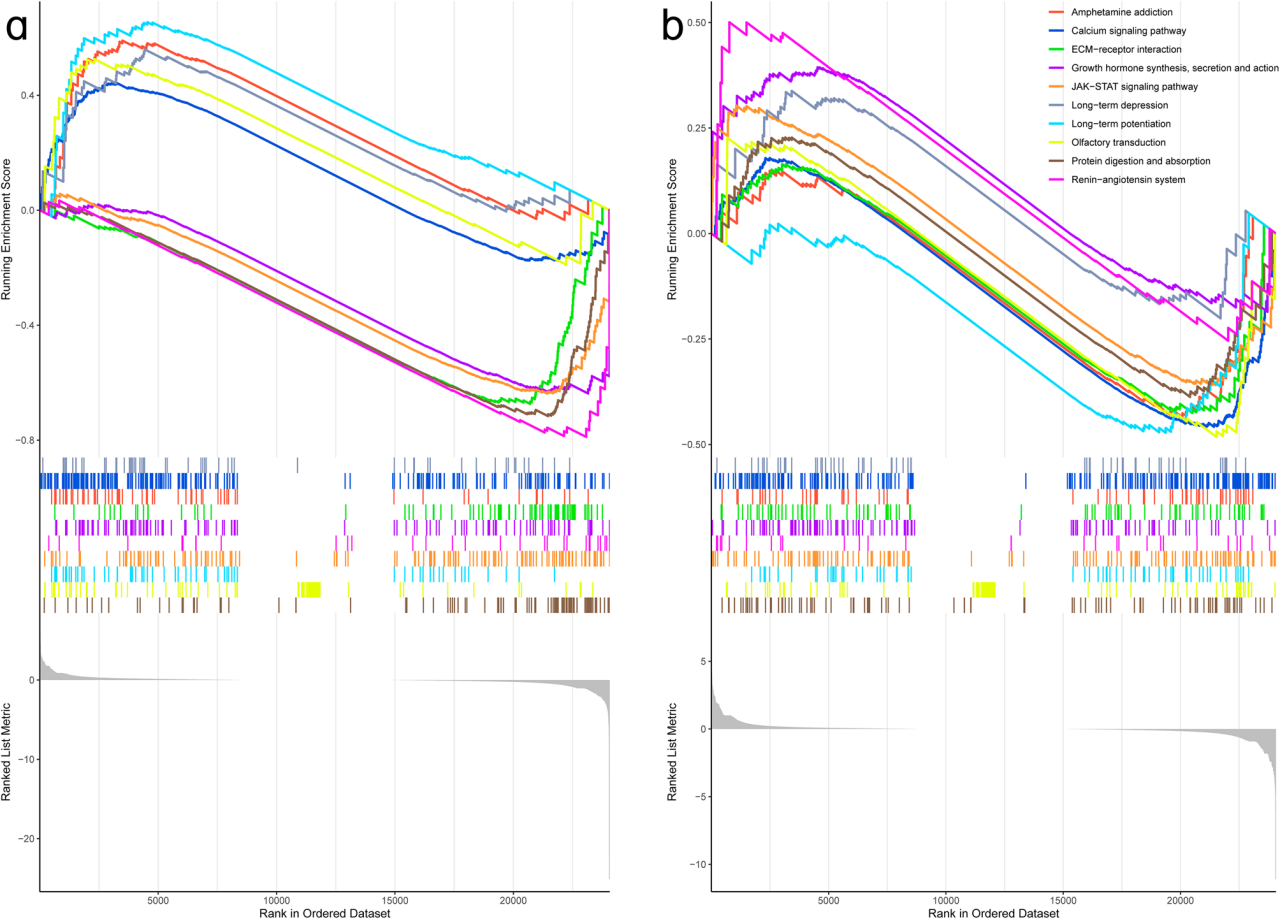
Enrichment analysis of GSEA pathways: (**a**) represents the top ten pathways that differ between groups B and C, with five pathways being up-regulated and five pathways being down-regulated. (**b**) Represents the transformation of A's ten paths between groups A and B.

The GSEA pathway enrichment analysis reveals 24 significant pathways between groups B and C. Supplement S3 shows GSEA enrichment between groups A and B and between groups B and C. The top ten pathways were depicted using enrichment maps. These ten pathways are also depicted in the enrichment diagrams for Groups A and B. Figure 7 shows that YWD can inhibit Long-term potentiation, Olfactory transduction, Amphetamine addiction, and the Calcium signaling pathway. Furthermore, it can stimulate growth hormone synthesis, secretion, and action and the Renin-angiotensin system (RAS) pathway.

### Network pharmacology

**Figure 8:**
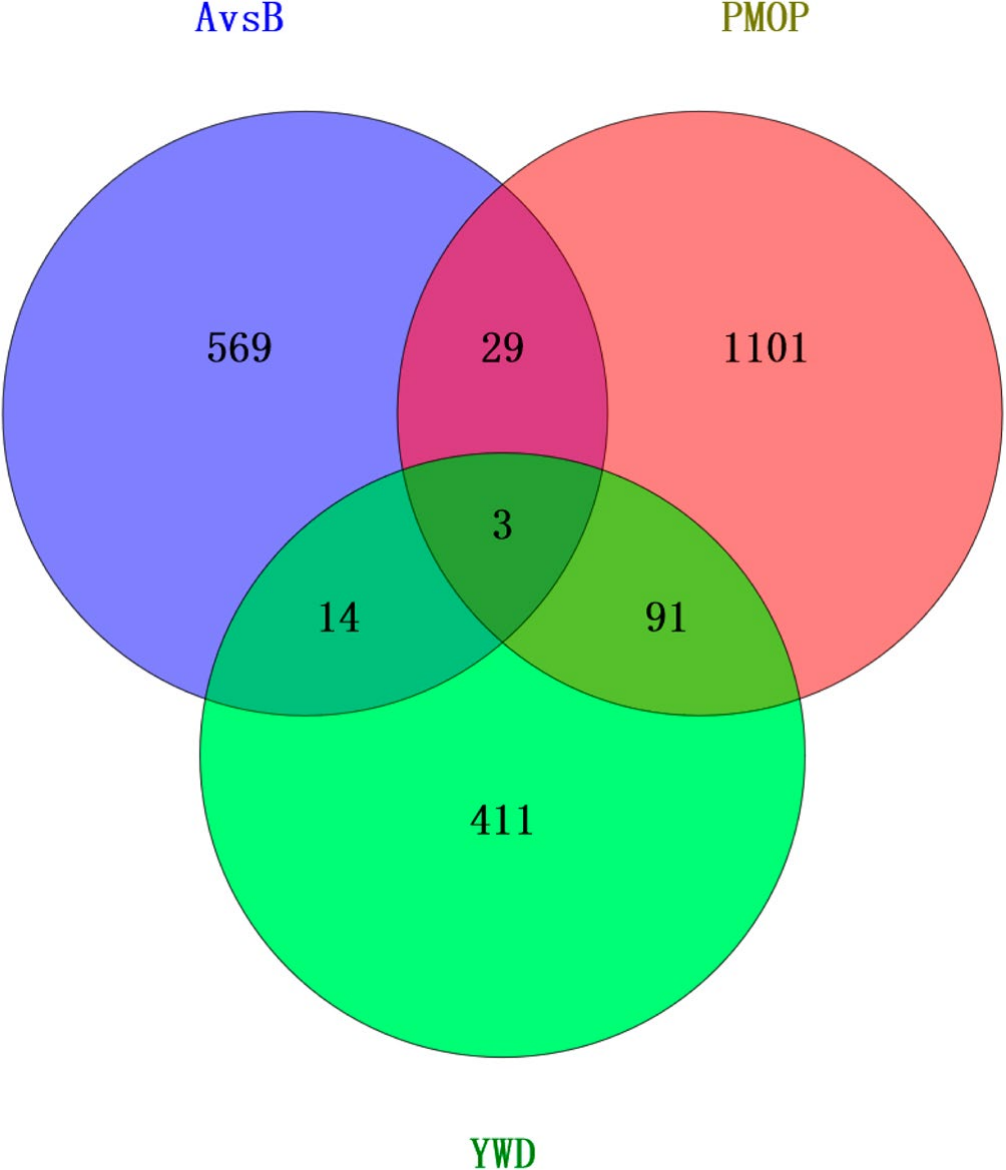
The intersection of the target Venny displays 108 AvsB and PMOP targets that can be acted on by YWD. Between AvsB and PMOP, there are 32 intersection targets.

**Figure 9:**
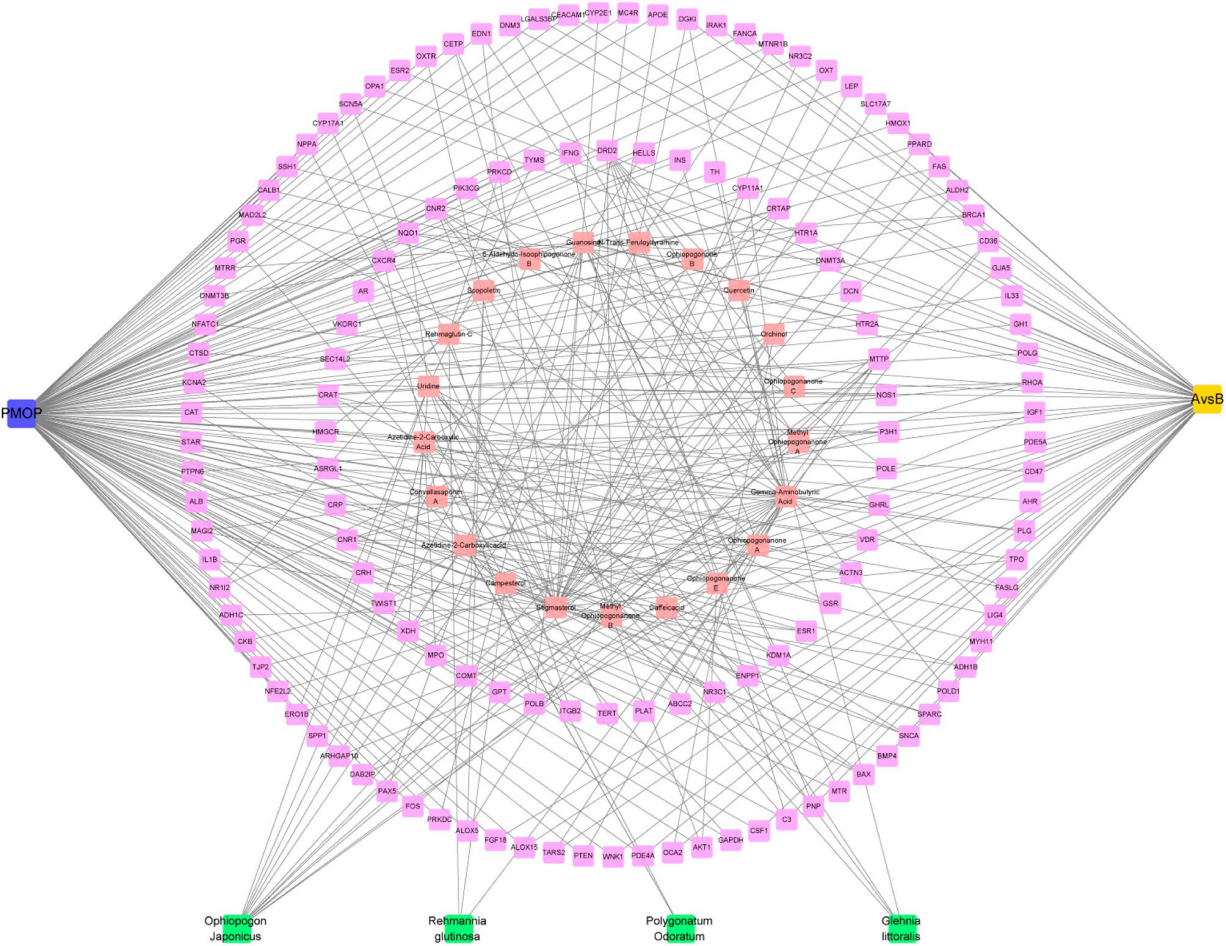
CHM network diagram with 137 targets, 4 TCM, and 22 active ingredients. Stigmasterol has the most targets of any active ingredient.

**Figure 10:**
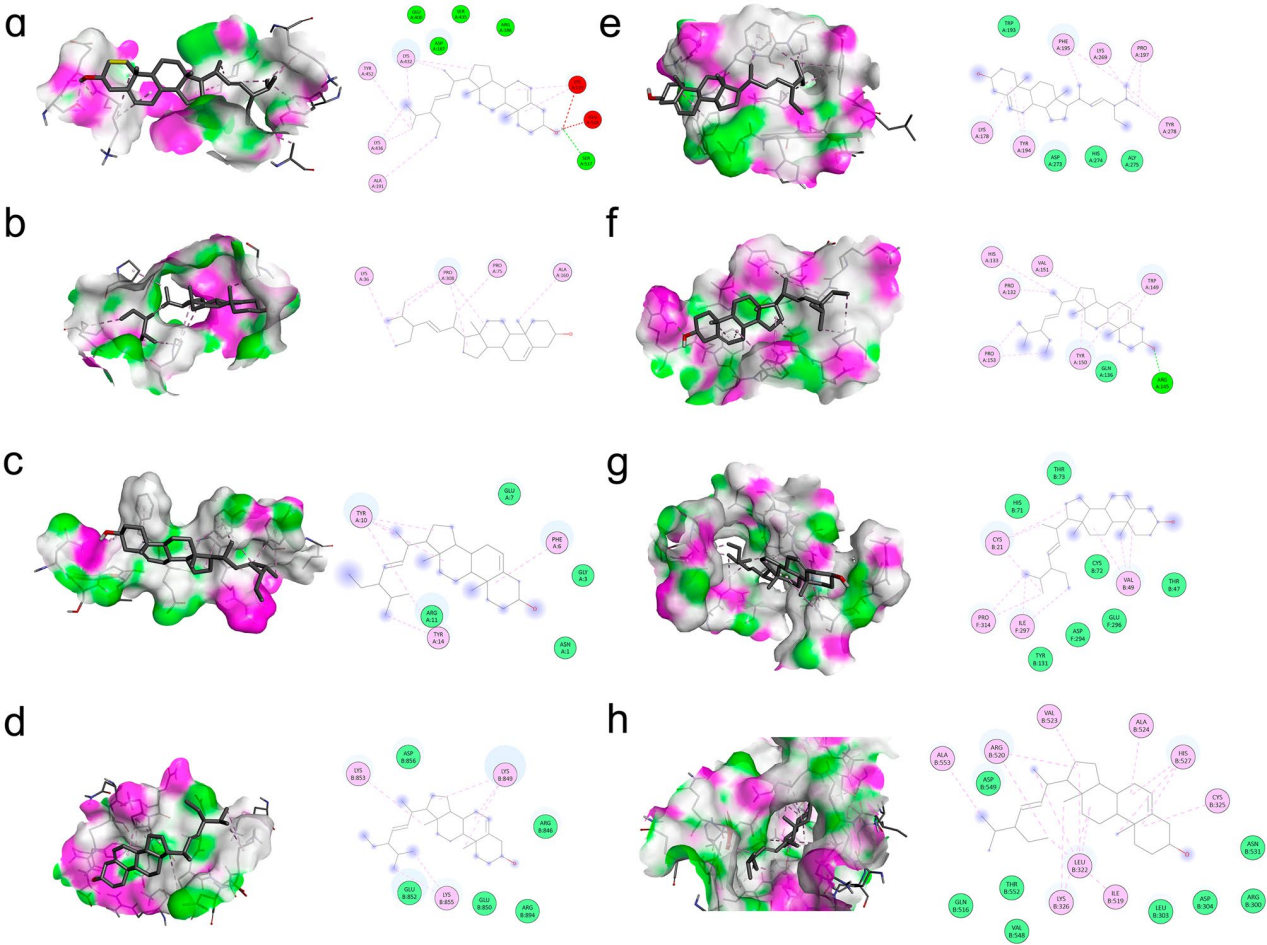
Details on molecular docking: (**a**) Stigmasterol-ALB; (**b**) Stigmasterol-CLTC; (**c**) Stigmasterol-CUL3; (**d**) Stigmasterol-EIF4G1; (**e**) Stigmasterol-KAT5; (**f**) Stigmasterol-KIF23; (**g**) Stigmasterol-RPL5; (**h**) Stigmasterol-VCL. The figure's left side of each molecular docking case shows the 3D structure, and the right side shows the 2D structure.

Figure 8 depicts 519 acting targets for YWD and 1224 associated targets for PMOP. Figure 9 depicts the CHM-active ingredient-target-disease network diagram of intersection targets among various parts, with Stigmasterol having the most targets. By using molecular docking, it was found that the top ten core targets of Degree in the protein interaction network were well docked with Stigmasterol, as detailed in Fig. 10. Online Appendix S4 provides information on the molecular binding energy, the protein source of binding, the docking box, and the spatial location. No docking structures were found for the two targets AKAP9 and SLC17A7.

**Figure 11:**
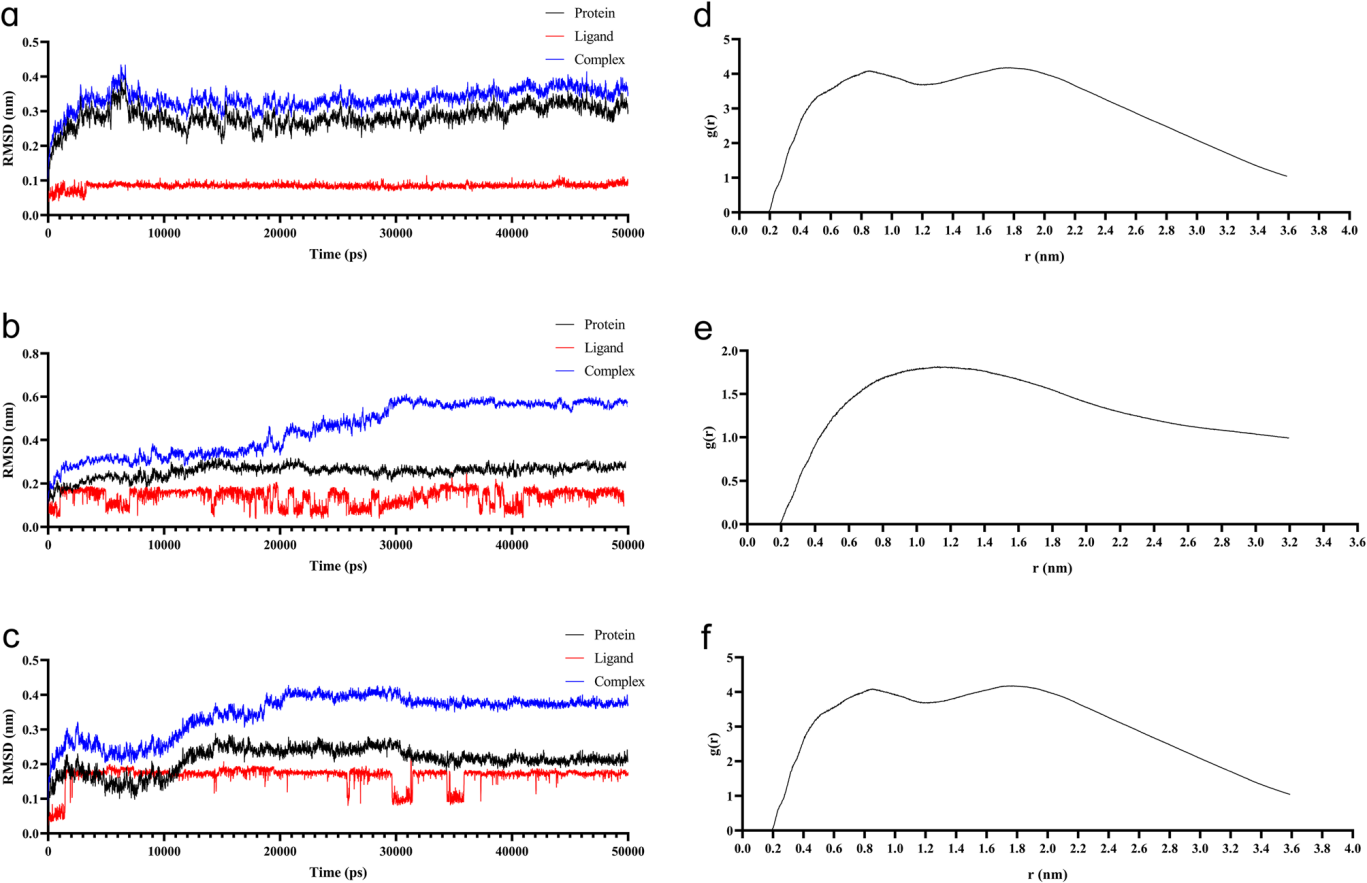
Results based on MD following 50 ns of running. Three docking RMSD plots are shown in (**a–c**). The protein, Stigmasterol, and the complex representing the protein and the small molecule are represented by the fluctuation curves in black, red, and blue, respectively. The binding becomes more unstable the more the curvature fluctuates. The three proteins' and molecules' RMSDs revealed that the complex's binding started to stabilize at 50 ns. (**a**) Stigmasterol-ALB, (**b**) Stigmasterol-KAT5 and (**c**) Stigmasterol-VCL. The RDF plots are shown in (**d**–**f**). (**d**) Stigmasterol-ALB, (**e**) Stigmasterol-KAT5 and (**f**) Stigmasterol-VCL.

Using molecular docking, Stigmasterol was discovered to bind well to the core proteins ALB, VCL, KAT5, and CLTC. For more information, see Fig. 10. The docking and binding of stigmasterol with ALB, KAT5 and VCL (the top three target proteins of Degree) were observed by molecular dynamics simulation. It was found that the root mean square deviation (RMSD) tended to be stable when running for 50 ns. Stigmasterol is stably bound to all three core proteins. RDF stands for the ratio of the average distance traveled by a molecule along a particular direction in a given amount of time to the initial distance, which can be used to represent the state of molecular motion. For details, see Fig. 11. The MD trajectory was used to calculate MMGBSA using the MMPBSA method, which more correctly depicts the target protein's interaction with the small molecule. Negative values show that the small molecule has a binding affinity for the target protein, and lower values show a stronger binding. According to the computed results, these small compounds have strong binding affinities to the associated proteins. Table 1 provides specifics on the binding energy.

**Table 1:** Binding free energy and energy value of each receptor-ligand complexes.

Receptor-ligand complex	Van der Waals (kJ mol^−1^)	Electrostatic potential energy (kJ mol^−1^)	Molecular mechanics/generalized born surface area (kJ mol^−1^)	Polar solvation energy (kJ mol^−1^)	Nonpolar solvation energy (kJ mol^−1^)	Molecular mechanical term energy (kJ mol^−1^)
Stigmasterol-ALB	− 27.80 ± 2.17	− 0.12 ± 0.70	− 16.20 ± 2.30	15.29 ± 1.19	− 3.57 ± 0.25	− 27.91 ± 2.42
Stigmasterol-KAT5	− 13.19 ± 1.4	− 0.05 ± 0.43	− 7.15 ± 0.91	7.83 ± 1.12	− 1.75 ± 0.25	− 13.23 ± 1.66
Stigmasterol-VCL	− 24.77 ± 2.01	− 3.47 ± 0.67	− 20.14 ± 2.11	11.36 ± 3.27	− 3.26 ± 0.29	− 28.24 ± 5.01

## Discussion

Estrogen is closely linked to female bone development and bone mass maintenance^20^. When a woman enters menopause, the level of estrogen in her body decreases, causing bone mass loss and changes in bone tissue structure, making bone brittle and prone to fracture, as well as pain, bone deformation, and fracture complications, all of which hurt on her health and quality of life^21,22^. Ovarian function declines prematurely in POI patients, causing estrogen levels to fall to menopausal levels. As a result, POI may result in an earlier onset of osteoporosis^23^. Furthermore, loss of ovarian function due to ovarian removal or cancer chemotherapy can result in the onset of osteoporosis at a young age^24^.

The hypothalamus is involved in the pathogenesis of osteoporosis. According to research, the hypothalamus is involved in bone metabolism in women with or without ovarian function loss^25^. Our study found that serum levels of GnRH and FSH were higher in POI rats than in normal rats, and that YWD could reduce serum levels of GnRH and FSH in POI rats. GnRH can stimulate the pituitary gland to release FSH. Studies have shown that perimenopausal women still suffer bone loss even when serum estrogen levels are normal^26^. This suggests that osteoporosis after ovarian loss is influenced by factors other than estrogen. FSH can promote bone resorption and osteoclast formation, as well as osteoporosis. In a cross-sectional study conducted in the United States, BMD was inversely related to serum FSH in premenopausal and early menopausal women^27^. This also suggests that the hypothalamus plays a role in the pathogenesis of osteoporosis.

Protein interaction was used to obtain key targets such as ALB, VCL, KAT5, and CLTC. By using molecular docking with MD, it was also confirmed that important components can bind to essential targets. ALB is a plasma calcium and magnesium transporter that may regulate plasma osmotic pressure^28^. VCL participates in cell chemical signal transduction and is important in cell adhesion, extension, movement, proliferation, survival, and other processes^29^. VCL is a promising marker for osteoporosis and bone loss diagnosis^30^. KAT5 is a chromatin-modifying enzyme under expressed in osteoporosis patients' osteoblasts^31^. CLTC is involved in bone loss in rheumatoid arthritis and is important in osteoclastic progenitor cell fusion^32^. Stigmasterol can be combined with ALB, VCL, KAT5, and CLTC after molecular docking, providing a new direction for future research. As shown in Fig. 8, our research discovered that YWD can act on 32 PMOP-related targets. C3, SNCA, and LIG4 were predicted targets of YWD active components. C3 is essential for the activation of the complement system. The complement system can regulate the function of osteoblasts and osteoclasts and may be involved in bone homeostasis regulation^33,34^. SNCA is a key regulator of bone homeostasis, and inhibiting it can help to prevent bone loss after oophorectomy^35,36^. LIG4 is a DNA ligase enzyme. When stem cells have LIG4 defects, genomic instability rises, the cell cycle is disrupted, and differentiation ability suffers^37^.

Our research has identified many potential research pathways. The JAK-STAT signaling pathway, for example, is involved in numerous important biological processes, including cell growth, differentiation, apoptosis, and immune regulation. According to growing evidence, the JAK/STAT signaling pathway is important in bone development, metabolism, healing, and aging^38,39^. Growth hormone promotes bone growth and, when deficient, increases osteoclast activity, which can cause bone damage^40^. Figure 7 shows that compared to group C, growth hormone synthesis, secretion, and action were down-regulated in group B, whereas YWD could up-regulate the pathway. In bone tissue, RAS regulates cell proliferation, differentiation, apoptosis, oxidative stress, inflammatory response, and angiogenesis^41,42^. Calcium ion is required for signal transduction in many cells and tissues and is involved in cell vitality, exocytosis, motility, apoptosis, and transcription^43^. The calcium signaling pathway is linked to osteoporosis^44^. Enriching KEGG and GSEA pathways suggests this pathway is an important target for YWD intervention in POI-related osteoporosis.

## Conclusion

Our study focused on the hypothalamus to investigate the potential mechanism of YWD in preventing osteoporosis in POI rats. We concluded that YWD can reduce GnRH and FSH in POI rats, with the key targets being ALB, C3, SNCA, and CLTC. Calcium signaling, growth hormone synthesis, secretion, and action, and the renin-angiotensin system are critical. However, more research is needed to confirm the specific roles of key targets and pathways.

### Supplementary Information

Supplementary Information 1.

Supplementary Information 2.

Supplementary Information 3.

Supplementary Information 4.

## Data Availability

All data included or relevant to the study are available upon request by contact with the corresponding author.

## Abbreviations

POI: Premature ovarian insufficiency
YWD: Yiwei Decoction
rRNA: Ribosomal RNA
CTX: Cyclophosphamide
CHM: Chinese herbal medicine
RAS: Renin-angiotensin system
